# Initial Insights into the Genetic Variation Associated with Metformin Treatment Failure in Youth with Type 2 Diabetes

**DOI:** 10.1155/2023/8883199

**Published:** 2023-05-24

**Authors:** Shylaja Srinivasan, Ling Chen, Miriam Udler, Jennifer Todd, Megan M. Kelsey, Morey W. Haymond, Silva Arslanian, Philip Zeitler, Rose Gubitosi-Klug, Kristen J. Nadeau, Katherine Kutney, Neil H. White, Josephine H. Li, James A. Perry, Varinderpal Kaur, Laura Brenner, Josep M. Mercader, Adem Dawed, Ewan R. Pearson, Sook-Wah Yee, Kathleen M. Giacomini, Toni Pollin, Jose C. Florez

**Affiliations:** 1 Division of Pediatric Endocrinology, University of California at San Francisco, San Francisco, CA, USA; 2 Center for Genomic Medicine and Diabetes Unit, Massachusetts General Hospital, Boston, MA, USA; 3 Department of Medicine, Harvard Medical School, Boston, MA, USA; 4 Programs in Metabolism and Medical & Population Genetics, Broad Institute of Harvard & Massachusetts Institute of Technology, Cambridge, MA, USA; 5 Division of Pediatric Endocrinology, University of Vermont, Burlington, VA, USA; 6 Division of Pediatric Endocrinology, University of Colorado School of Medicine, Aurora, CO, USA; 7 Department of Pediatrics, Baylor College of Medicine, Houston, TX, USA; 8 UPMC Children’s Hospital of Pittsburgh, Departments of Pediatrics, University of Pittsburgh School of Medicine, Pittsburgh, PA, USA; 9 Division of Pediatric Endocrinology and Metabolism, Case Western Reserve University and Rainbow Babies and Children’s Hospital, Cleveland, OH, USA; 10 Division of Endocrinology, Metabolism & Lipid Research, Washington University School of Medicine, St Louise, MO, USA; 11 Department of Medicine, University of Maryland School of Medicine, Baltimore, MD, USA; 12 Population Health & Genomics, School of Medicine, University of Dundee, Dundee, UK; 13 Department of Bioengineering and Therapeutics, University of California, San Francisco, CA, USA

## Abstract

Metformin is the first-line treatment for type 2 diabetes (T2D) in youth but with limited sustained glycemic response. To identify common variants associated with metformin response, we used a genome-wide approach in 506 youth from the Treatment Options for Type 2 Diabetes in Adolescents and Youth (TODAY) study and examined the relationship between T2D partitioned polygenic scores (pPS), glycemic traits, and metformin response in these youth. Several variants met a suggestive threshold (*P* < 1 × 10^−6^), though none including published adult variants reached genome-wide significance. We pursued replication of top nine variants in three cohorts, and rs76195229 in *ATRNL1* was associated with worse metformin response in the Metformin Genetics Consortium (*n* = 7,812), though statistically not being significant after Bonferroni correction (*P* = 0.06). A higher *β*-cell pPS was associated with a lower insulinogenic index (*P* = 0.02) and C-peptide (*P* = 0.047) at baseline and higher pPS related to two insulin resistance processes were associated with increased C-peptide at baseline (*P* = 0.04,0.02). Although pPS were not associated with changes in glycemic traits or metformin response, our results indicate a trend in the association of the *β*-cell pPS with reduced *β*-cell function over time. Our data show initial evidence for genetic variation associated with metformin response in youth with T2D.

## 1. Introduction

The incidence of type 2 diabetes (T2D) in youth is increasing in the United States and worldwide [1, 2]. Youth with T2D have an aggressive disease course with early onset and severe burden of complications [3]. Metformin is currently the foundation of treatment of T2D and remains one of the few FDA-approved options in addition to insulin and glucagon-like peptide receptor agonists for the management of T2D in youth. However, despite a good initial response [4, 5], over time, youth with T2D have poorer responses to metformin than those observed in adults. For example, despite initial good responses, 52% of the youth participants in the Treatment Options for Type 2 Diabetes in Adolescents and Youth (TODAY) study failed to have a sustained glycemic response to metformin therapy [6], whereas only 12% of adults with T2D in the ADOPT study failed after the same duration of metformin treatment [7]. Understanding reasons for variations in response to metformin is needed to characterize individuals into likely responders and non-responders and to shed further light on the mechanism(s) of action underlying metformin response in youth.

Using the genome-wide complex trait analysis method, the heritability of metformin response is estimated to explain a substantial proportion (21–34%) of the variation in metformin response depending on how glycemic response is measured [8]. Indeed, genome-wide association studies (GWAS) have revealed loci associated with metformin response in adults with established T2D as well as in adults at high risk for T2D [9–12]. However, the genetic determinants of metformin response in youth remain unexplored. Our objective was to evaluate the genetic determinants of metformin failure in youth through a genome-wide approach by searching for novel variants and examining the effect of known genetic variants associated with metformin response in adults. A secondary objective was to evaluate the biological mechanisms underlying metformin response using partitioned polygenic scores (pPS) derived from genetic clustering of T2D loci.

## 2. Methods

### 2.1. Description of Participants.

This study was undertaken by the Progress in Diabetes Genetics in Youth (ProDiGY) consortium [13], a collaboration of the TODAY [6], SEARCH for Diabetes in Youth [14], and T2D-GENES [15] study groups. We examined genetic determinants of metformin response in 506 youth with T2D from the TODAY study, after excluding participants with monogenic diabetes (*n* = 22) [16, 17]. The design and results of the TODAY study have been previously described [6], with the primary outcome being loss of glycemic control, defined as a HbA1c ≥ 8% for 6 months, or sustained metabolic decompensation requiring insulin. Of note, the American Indian Tribal Nations that partnered with the TODAY study elected not to participate in the genomics collection [18].

### 2.2. Genotyping, Imputation, and Quality Control.

Samples were genotyped on the Infinium array by the Genomics Platform at the Broad Institute. Genotypes were called using the autocall algorithm with quality control steps run in PLINK2 and R-3.4. Imputation was performed using the TOPMed Imputation Server against the TOPMed r2 panel as the reference, with the imputation threshold (*R*^2^) set at 0.5, yielding 24,813,350 autosomal single nucleotide polymorphisms (SNPs) for analysis.

### 2.3. Construction of Partitioned Polygenic Scores (pPS).

The methods to construct the pPS have been previously described [19]. Briefly, a soft-clustering approach was used on 94 genetic variants associated with T2D risk and 47 diabetes-related traits to create five pPS, namely, two clusters representing reduced *β*-cell function, differing from each other by high versus low proinsulin levels and three other clusters that displayed features of insulin resistance, namely, (1) obesity-mediated, (2) “lipodystrophy-like” fat distribution, and (3) disrupted liver and lipid metabolism. In TODAY, individual pPS was constructed for each participant by multiplying the number of risk alleles present per SNP by the cluster weight reported for that SNP and then summing the results over the SNPs.

### 2.4. Replication Analyses.

An evaluation of top findings (*P* < 1 × 10^−6^) was conducted within three adult cohorts: the Metformin Genetics Consortium (MetGen) [10], the Diabetes Prevention Program (DPP) [20], and the Study to Understand the Genetics of the Acute Response to Metformin and Glipizide in Humans (SUGAR-MGH) [21]. These cohorts have independently performed GWAS for metformin response [10, 22, 23]. A total of 10 lookups were done, and each SNP was evaluated for association with metformin response based on the outcomes and covariates used in the respective GWAS (Supplementary Table 2). Binomial tests were performed to compare the effects of the top SNPs associated with metformin response in ProDiGY with data from MetGen, DPP, and SUGAR-MGH.

### 2.5. Statistical Analysis.

Genome-wide analysis of time to metformin treatment failure was run under a Cox proportional hazards model in gwasurvivr (an R package) using an additive genetic model, adjusting for age, sex, top three principal components (PCs), and treatment arms (metformin alone, metformin + rosiglitazone, and metformin + lifestyle), similar to the prior analyses in the TODAY study [6]. For the pPS analyses, general linear models were used to test association with glycemic traits and change in traits over 6 months. The association between pPS and treatment failure as defined by TODAY was tested using a Cox proportional hazards model. The cluster analyses were adjusted for age, sex, first three PCs, and treatment arms and were run in R-4.0.

## 3. Results

### 3.1. Baseline Demographics.

**Figure 1:**
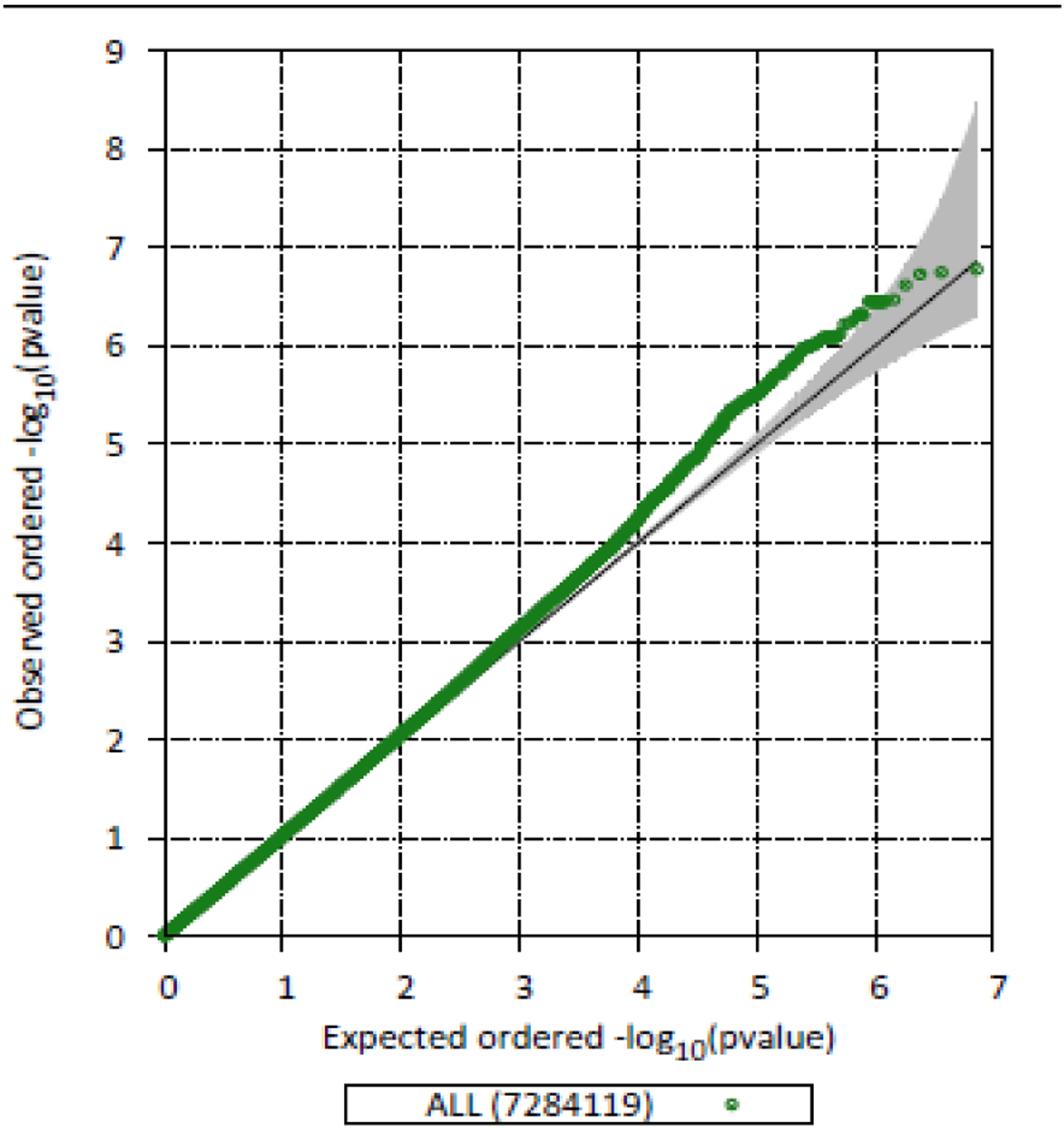
Quantile-quantile plot. The *X* axis shows the expected distribution and the *Y* axis shows the observed distribution of findings. *λ*_GC_ = 1.09.

The demographics of the 506 TODAY participants at baseline are summarized in Table 1. The mean age was 14 ± 2 years, 65% were female, and the mean BMI *Z*-score was 2.23 ± 0.5. The majority of participants were youth of color with 20% identifying as non-Hispanic White, 37% as non-Hispanic Black, and 35% as Hispanic. Mean HbA1c at the end of the run-in period and prerandomization was 6.0 ± 0.7%. The quantile-quantile plot is shown in Figure 1 and *λ*_GC_ was 1.09, filtering for a minor allele frequency of 5%.

### 3.2. Genome-Wide Association Testing.

**Figure 2:**
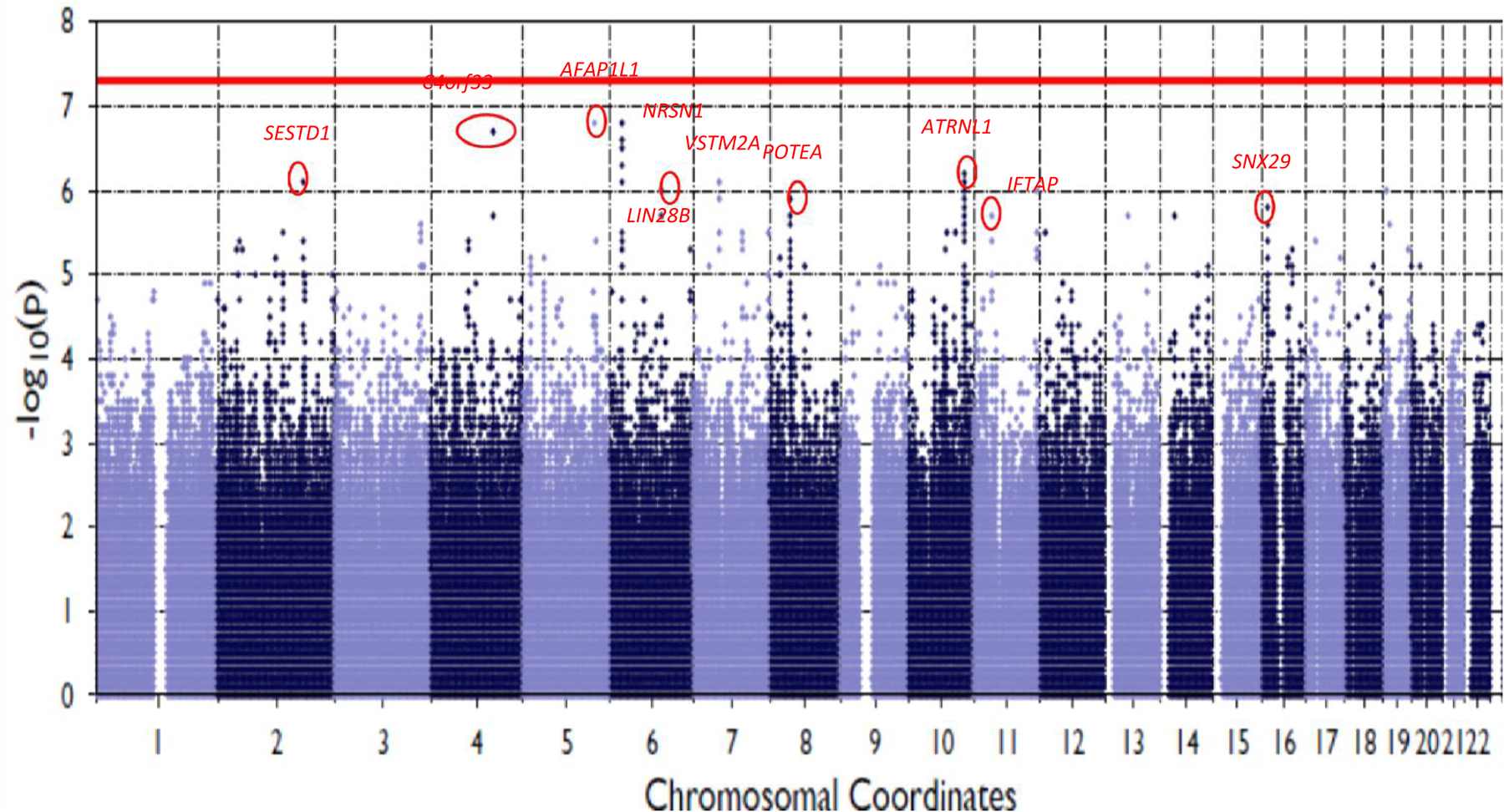
Manhattan plot with the top associations is highlighted (*P* < 1 × 10^−6^). The horizontal line in the plot indicates the genome wide significance (*P*) value threshold of 5 ×10^−8^.

Several genetic variants (*n* = 10) met a suggestive significance threshold of *P* < 1 × 10^−6^, though none reached genome-wide significance (Manhattan plot is shown in Figure 2). Top findings are shown in Supplementary Table 1.

### 3.3. Replication Analyses.

Given the modest sample size, top findings were examined across the cohorts of MetGen (*n* = 7,812), DPP (*n* = 1,763), and SUGAR-MGH (*n* = 807) where metformin response has been defined in adults (results in Supplementary Table 3). rs76195229 in an intron of *ATRNL1* was significantly associated with worse metformin response (*β* = 0.336 ± 0.125, *P* = 0.007) in MetGen where the outcome was glycemic response, as measured by baseline minus minimum on-treatment HbA1c within 18 months after metformin initiation. However, when accounting for the nine variants that were evaluated, the findings were no longer statistically significant (*P* = 0.06). Our top variants were not significant in the DPP or SUGAR-MGH cohorts. Binomial tests to compare the top variants in ProDiGY with the replication cohorts showed that 70% (*P* = 0.34), 90% (*P* = 0.02), and 60% (*P* = 0.75) of the SNPs had the same direction of effect in the MetGen, SUGAR-MGH, and DPP cohorts, respectively. We also performed lookups of published variants associated with metformin response in adults as well as variants associated with metformin transporters (Supplementary Table 4) and did not find any associations at *P* < 0.05.

### 3.4. Genetic Cluster Analyses.

For quality control, we examined the association of the pPS for each of the five T2D genetic clusters with select metabolic traits and the results were in the expected direction and similar to findings in adults [19, 24] (Supplementary Table 5). The associations between pPS and quantitative glycemic traits at baseline are shown in Table 2. A higher *β*-cell cluster score was significantly associated with a lower insulinogenic index and C-peptide. For the clusters representing features of insulin resistance, higher lipodystrophy and liver/liver pPS were associated with increased C-peptide levels. The association between pPS and change in glycemic traits from baseline to 6 months were not significant, but there was a trend in the association of the *β*-cell cluster, worsening C-peptide index over time (Supplementary Table 6). The associations between pPS and metformin response using the Cox proportional hazards model were not significant (Supplementary Table 6).

## 4. Discussion

To our knowledge, this is the first large-scale evaluation of the genetics of metformin response in youth with T2D. Though we did not identify any genome-wide significant findings, we were able to identify several associations that met a suggestive threshold. As participants were subject to a run-in period and needed to maintain HbA1c of <8% on metformin monotherapy for randomization, it is possible that the run-in period excluded those with the poorest response to metformin and removed some variation within the sample, thus reducing power. We also validated pPS derived from genetic clustering of T2D loci in our youth-onset T2D population, based on associations with glycemic and metabolic traits that were consistent with associations observed in adults.

Although our study represents the largest existing genetic dataset for youth with T2D, our sample size was modest. We therefore chose to evaluate our top findings in adult cohorts with well-defined metformin response. We show a trend towards significance for association between rs76195229 and metformin response in adults from MetGen, the largest meta-analysis evaluating glycemic response to metformin in adults with T2D [10]. rs76195229 is an intronic variant in the *ATRNL1* (attractin like 1) gene on chromo-some 10 and is predicted to be associated with carbohydrate binding. According to the UniProt Knowledgebase, *ATRNL1* may influence melanocortin signaling in pathways that regulate energy homeostasis. In MetGen, individuals who were homozygous for this variant had a 0.34% higher HbA1c on metformin compared to those with the wild-type allele. In addition to rs76195229, several of our top results have associations with glycemic and metabolic traits. As an example, rs10040292 in the *AFAP1L1* intron is associated with waist-hip-ratio in the GIANT-UK Biobank GWAS meta-analyses (*P* = 8.7 × 10^−7^) and with insulin sensitivity in GENESIS GWAS (*P* = 0.005). A complete list of associated traits for our top findings is listed in Supplementary Table 1. These findings merit exploration in other pediatric cohorts. We could not confirm the reported genetic associations influencing glycemic response to metformin that have been found in adults, either because our sample size was not large enough to detect these associations based on the reported effect sizes or because genetic variation may influence metformin response differently in youth compared to adults with T2D. Another factor to consider is adherence to metformin which has been shown to be worse in younger populations compared to adults [18]. Our data here are from the original TODAY clinical trial where there was frequent contact with participants and where medication adherence was greater than 70% across all treatment arms and not found to be a factor associated with metformin treatment failure [25].

In the cluster analyses, a greater cluster score for *β*-cell function was associated with lower baseline *β*-cell function and a trend towards reduced *β*-cell function over time. This is similar to results observed in adults at risk for diabetes in the Diabetes Prevention Program, where a high *β*-cell pPS was associated with an increased risk of diabetes and worsening in insulin secretion despite interventions with intensive lifestyle and metformin [26]. In the future, analyses of process-specific genetic clusters, particularly when combined with clinical phenotyping, could offer additional insight on the mechanisms of disease and drug response.

While studies in adults with T2D have shown variants associated with metformin response, there are virtually no such studies in youth to date. Youth in the TODAY study who were subsequently found to have HNF4A Maturity Onset Diabetes of the Young (MODY) were more likely to experience glycemic failure on metformin, a finding that was not surprising given their expected preferential response to sulfonylureas [17]. A study of 124 children with obesity randomized to either metformin or placebo for weight loss over a 6-month period conducted post hoc genotyping for 225 candidate SNPs previously associated with obesity or metformin pharmacogenetics. The authors did not identify any statistically significant associations of the chosen variants with weight change on metformin, but there was a trend towards significance for 28 common variants including novel variants in *ADYC3* and *BDNF* which were associated with worse response and improved response, respectively [27].

Strengths of our study include the detailed phenotyping and longitudinal characterization of metformin response in the TODAY study. Additionally, our cohort was multiethnic and truly representative of youth-onset T2D with the majority of participants being youth of color. Lastly, in ProDiGY, we have established the largest known genetic dataset for youth-onset T2D that can be meta-analyzed with future studies, as the burden of youth-onset T2D continues to increase [2, 28]. We attempted to counter the modest sample size for genetic analyses with validation in three independent cohorts and through lookups of all variants associated with metformin response in adults. Additional limitations include the different definitions of metformin response in the replication cohorts, the white European predominance of the MetGen dataset, and the exclusion of metformin failures during run-in in TODAY.

In conclusion, we have generated a resource that may help prioritize genetic determinants of metformin response in youth with T2D from the TODAY study. As the burden of T2D in youth continues to increase, pediatric clinical studies should prioritize collection of genetic data so that future studies are sufficiently powered to detect significant associations.

## Supplementary Material

Supplementary Material

## Figures and Tables

**Table 1:** Baseline demographics of TODAY participants.

Characteristics	
*n* =	506
Age (years) (mean ± SD)	14.44 ± 1.99
Female (%)	64.62
Race/ethnicity *n* (%)	
Hispanic or latino	178 (35.2)
Non-hispanic black	185 (36.6)
Non-hispanic white	99 (19.5)
Other	44 (8.7)
BMI *Z* score (mean ± SD)	2.23 ± 0.46
Fasting glucose (mg/dL) (mean ± SD)	147.65 ± 52.36
Fasting insulin (*μ*U/mL) (mean ± SD)	32.91 ± 21.68
HbA1c % (mean ± SD)	6.02 ± 0.74

**Table 2:** Association of the 5 partitioned polygenic scores with baseline quantitative glycemic traits.

	*β*-cell		Proinsulin		Obesity		Lipodystrophy		Liver/lipid	
	*β*	*P* value	*β*	*P* value	*β*	*P* value	*β*	*P* value	*β*	*P* value
ln fasting glucose	0.002	0.27	0.0002	0.96	−0.003	0.23	0.003	0.27	0.001	0.7
ln fasting insulin	−0.01	0.07	−0.007	0.62	0.006	0.38	0.008	0.26	0.013	0.13
C peptide	−0.026	0.047	−0.02	0.54	0.007	0.71	0.04	0.04	0.05	0.02
Insulin sensitivity index	0.01	0.1	0.0066	0.62	−0.007	0.34	−0.009	0.24	−0.015	0.092
Insulinogenic index	−0.02	0.02	0.007	0.74	0.021	0.066	−0.004	0.76	0.01	0.5
C-peptide index	−0.013	0.08	0.0023	0.9	0.011	0.24	0.0063	0.51	0.014	0.24
Oral disposition index	−0.005	0.49	0.011	0.56	0.014	0.18	−0.001	0.94	−0.006	0.6

## Data Availability

The dataset analyzed in the current study is available at dbGap (dbGaP Study Accession: phs001511.v1.p1, https://www.ncbi.nlm.nih.gov/projects/gap/cgi-bin/study.cgi?study_id=phs001511.v1.p1).

## References

[1] Pinhas-Hamiel O and Zeitler P, “The global spread of type 2 diabetes mellitus in children and adolescents,” The Journal of Pediatrics, vol. 146, pp. 693–700, 2005. 15870677 10.1016/j.jpeds.2004.12.042

[2] Mayer-Davis EJ, Lawrence JM, Dabelea D , “Incidence trends of type 1 and type 2 diabetes among youths, 2002–2012,” New England Journal of Medicine, vol. 376, pp. 1419–1429, 2017. 28402773 10.1056/NEJMoa1610187 PMC5592722

[3] Today Study Group, “Long-term complications in youth-onset type 2 diabetes,” New England Journal of Medicine, vol. 385, no. 5, pp. 416–426, 2021. 34320286 10.1056/NEJMoa2100165 PMC8697255

[4] Kelsey MM, Geffner ME, Guandalini C , “Presentation and effectiveness of early treatment of type 2 diabetes in youth: lessons from the TODAY study,” Pediatric Diabetes, vol. 17, pp. 212–221, 2016. 25690268 10.1111/pedi.12264 PMC4539288

[5] Laffel L, Chang N, Grey M , “Metformin monotherapy in youth with recent onset type 2 diabetes: experience from the prerandomization run-in phase of the TODAY study,” Pediatric Diabetes, vol. 13, pp. 369–375, 2012. 22369102 10.1111/j.1399-5448.2011.00846.x PMC3371323

[6] TODAY Study Group, Zeitler P, Hirst K , “A clinical trial to maintain glycemic control in youth with type 2 diabetes,” New England Journal of Medicine, vol. 366, pp. 2247–2256, 2012. 22540912 10.1056/NEJMoa1109333 PMC3478667

[7] Nadeau KJ, Anderson BJ, Berg EG , “Youth-onset type 2 diabetes consensus report: current status, challenges, and priorities,” Diabetes Care, vol. 39, pp. 1635–1642, 2016. 27486237 10.2337/dc16-1066 PMC5314694

[8] Zhou K, Donnelly L, Yang J , “Heritability of variation in glycaemic response to metformin: a genome-wide complex trait analysis,” Lancet Diabetes & Endocrinology, vol. 2, pp. 481–487, 2014. 24731673 10.1016/S2213-8587(14)70050-6 PMC4038749

[9] GoDARTS and UKPDS Diabetes Pharmacogenetics Study Group, Wellcome Trust Case Control Consortium 2, Zhou K , “Common variants near ATM are associated with glycemic response to metformin in type 2 diabetes,” Nature Genetics, vol. 43, pp. 117–120, 2011. 21186350 10.1038/ng.735 PMC3030919

[10] Zhou K, Yee SW, Seiser EL , “Variation in the glucose transporter gene SLC2A2 is associated with glycemic response to metformin,” Nature Genetics, vol. 48, pp. 1055–1059, 2016. 27500523 10.1038/ng.3632 PMC5007158

[11] Rotroff DM, Yee SW, Zhou K , “Genetic variants in CPA6 and PRPF31 are associated with variation in response to metformin in individuals with type 2 diabetes,” Diabetes, vol. 67, pp. 1428–1440, 2018. 29650774 10.2337/db17-1164 PMC6014560

[12] Li JH, Brenner LN, Kaur V , “Genome-wide association analysis identifies ancestry-specific genetic variation associated with medication response in the Study to Understand the Genetics of the Acute Response to Metformin and Glipizide in Humans (SUGAR-MGH),” medRxiv, 2022, https://www.medrxiv.org/content/medrxiv/early/2022/01/24/2022.01.24.22269036.full.pdf. 10.1007/s00125-023-05922-7 PMC10790310 37233759

[13] Srinivasan S, Chen L, Todd J , “Te first genome-wide association study for type 2 diabetes in youth: the progress in diabetes genetics in youth (ProDiGY) consortium,” Diabetes, vol. 70, pp. 996–1005, 2021, https://diabetes.diabetesjournals.org/content/diabetes/70/4/996.full.pdf. 33479058 10.2337/db20-0443 PMC7980197

[14] SEARCH Study Group, “SEARCH for Diabetes in Youth: a multicenter study of the prevalence, incidence and classification of diabetes mellitus in youth,” Controlled Clinical Trials, vol. 26, pp. 458–471, 2004. 10.1016/j.cct.2004.08.002 15465616

[15] Fuchsberger C, Flannick J, Teslovich TM , “The genetic architecture of type 2 diabetes,” Nature, vol. 536, pp. 41–47, 2016. 27398621 10.1038/nature18642 PMC5034897

[16] Todd JN, Kleinberger JW, Zhang H , “Monogenic diabetes in youth with presumed type 2 diabetes: results from the progress in diabetes genetics in youth (ProDiGY) collaboration,” Diabetes Care, vol. 44, no. 10, pp. 2312–2319, Article ID 210491, 2021. 34362814 10.2337/dc21-0491 PMC8929184

[17] Kleinberger JW, Copeland KC, Gandica RG , “Monogenic diabetes in overweight and obese youth diagnosed with type 2 diabetes: the TODAY clinical trial,” Genetics in Medicine, vol. 20, pp. 583–590, 2018. 29758564 10.1038/gim.2017.150 PMC5955780

[18] Chadwick JQ, Copeland KC, Branam DE , “Genomic Research and American Indian tribal communities in Oklahoma: learning from past Research misconduct and building future trusting partnerships,” American Journal of Epidemiology, vol. 188, pp. 1206–1212, 2019. 31081852 10.1093/aje/kwz062 PMC6601530

[19] Udler MS, Kim J, von Grotthuss M , “Type 2 diabetes genetic loci informed by multi-trait associations point to disease mechanisms and subtypes: a soft clustering analysis,” PLoS Medicine, vol. 15, Article ID 1002654, 2018. 10.1371/journal.pmed.1002654 PMC6150463 30240442

[20] Knowler WC, Barrett-Connor E, Fowler SE , “Reduction in the incidence of type 2 diabetes with lifestyle intervention or metformin,” New England Journal of Medicine, vol. 347, pp. 393–403, 2002. 10.1056/NEJMoa012512 PMC1370926 11832527

[21] Walford GA, Colomo N, Todd JN , “The study to understand the genetics of the acute response to metformin and glipizide in humans (SUGAR-MGH): design of a pharmacogenetic resource for type 2 diabetes,” PLoS One, vol. 10, Article ID 121553, 2015. 10.1371/journal.pone.0121553 PMC4374872 25812009

[22] Li JH, Perry JA, Jablonski KA , “Identification of genetic variation influencing metformin response in a multi-ancestry genome-wide association study in the diabetes prevention Program (DPP),” Diabetes, Article ID 220702, 2022. 10.2337/db22-0702 PMC10382652 36525397

[23] Li JHBL, Kaur V, Figueroa K , “Genome-wide association analysis identifies ancestry-specific genetic variation associated with acute response to metformin and glipizide in SUGAR-MGH,” Diabetologia, 2022. 10.1007/s00125-023-05922-7 PMC10790310 37233759

[24] DiCorpo D, LeClair J, Cole JB , “Type 2 diabetes partitioned polygenic scores associate with disease outcomes in 454,193 individuals across 13 cohorts,” Diabetes Care, vol. 45, no. 3, pp. 674–683, 2022. 35085396 10.2337/dc21-1395 PMC8918228

[25] Katz LL, Anderson BJ, McKay SV , “Correlates of medication adherence in the TODAY cohort of youth with type 2 diabetes,” Diabetes Care, vol. 39, pp. 1956–1962, 2016. 27352955 10.2337/dc15-2296 PMC5079608

[26] Billings LK, Jablonski KA, Franks PW , “249-OR: type 2 diabetes (T2D) genetic clusters for association with glycemic responses to intervention for diabetes prevention,” Diabetes, vol. 70, no. 1, p. 70, 2021.

[27] Anguita-Ruiz A, Pastor-Villaescusa B, Leis R , “Common variants in 22 genes regulate response to metformin intervention in children with obesity: a pharmacogenetic study of a randomized controlled trial,” Journal of Clinical Medicine, vol. 8, no. 9, p. 1471, 2019. 31527397 10.3390/jcm8091471 PMC6780549

[28] Magge SN, Wolf RM, Pyle L , “The coronavirus disease 2019 pandemic is associated with a substantial rise in frequency and severity of presentation of youth-onset type 2 diabetes,” The Journal of Pediatrics, vol. 251, pp. 51–59, 2022. 35985535 10.1016/j.jpeds.2022.08.010 PMC9383958

