# Supplementary material for: Initial Insights into the Genetic Variation Associated with Metformin Treatment Failure in Youth with Type 2 Diabetes
Source: Pediatr Diabetes. 2023 May 24;2023:8883199. doi: 10.1155/2023/8883199 (PMC11000826; doi:10.1155/2023/8883199)
Supplement: Supplementary file 1 [file 8883199.f1.pdf]

Table 1: Nominally significant GWAS findings with a P value < 1 x 10-6

#CHROM	BEG	END
5	149321776	149321776
4	129436566	129436566
6	24058036	24058036
10	115789989	115789989
7	53995198	53995198
2	179285708	179285708
6	104958449	104958449
8	43596695	43596695
16	12539602	12539602

MARKER_ID	Effect_allele	*P* value
chr5:149321776:C:T	T	1.65E-07
chr4:129436566:C:T	T	1.83E-07
chr6:24058036:C:G	G	2.39E-07
chr10:115789989:A:G	G	5.79E-07
chr7:53995198:C:G	G	7.35E-07
chr2:179285708:C:T	T	8.32E-07
chr6:104958449:C:T	T	9.55E-07
chr8:43596695:C:T	T	1.17E-06
chr16:12539602:C:T	T	1.62E-06

Hazard ratio (HR) for metformin failure	HR lower 95% CI
0.42	0.3
0.5	0.39
0.4	0.29
0.46	0.34
0.5	0.39
0.53	0.41
0.49	0.36
0.44	0.32
0.52	0.4

HR upper 95% CI	MAF
0.58	0.0625
0.65	0.1062
0.57	0.0546
0.62	0.0685
0.66	0.0982
0.68	0.1687
0.65	0.0734
0.62	0.0635
0.68	0.0942

rs#	Nearest gene
rs10040292	AFAP1L1 intron
rs9996721	Close to C4orf33
rs9358746	Close to NRSN1
rs76195229	ATRNL1 intron (Interacts with MC4R)
rs4947856	Close to VSTM2A
rs1388182	Close to SESTD1
rs76236717	LIN28B intron
rs115911409	Close to POTEA
rs11645654	SNX29 intron

Top associated trait on T2D knowledge portal

Waist-hip ratio adj BMI, p-value=8.67e-6, beta=-0.0414 in GIANT-UK Biobank GWAS Meta-analysis
Urinary sodium excretion, p-value=0.0003, beta=-0.0064 in UK Biobank urinary sodium and potassium excretion
Iron deficiency, p-value=0.001093, OR=1.1923 in GERA additive model age-related GWAS
Creatine kinase, p-value=0.02407, beta=-0.0467 in several datasets
eGFR-cys (serum cystatin C), p-value=0.000486, beta=-0.0134 in CKDGen 1000G GWAS-eGFRcys associations
Left ventricular ejection fraction, p-value=0.0046, beta=-0.0445 in UK Biobank Cardiac MRI LV GWAS
Heart failure, p-value=0.007144, OR=1.0364 in several datasets
Uric acid, p-value=0.0004135, beta=-0.1416 in Middle Eastern Qatari population WGS
Coronary artery disease, p-value=0.0008661, OR=0.9774 in several datasets

Related trait on portal

Insulin sensitivity, p-value=0.005159, beta=0.4433 in GENESIS GWAS
Fasting insulin adj BMI, p-value=0.05971, beta=0.0242 in several datasets
Disposition index adj BMI, p-value=0.05173, beta=-0.074 in IVGTT-Based Insulin Secretion GWAS
Disposition index adj BMI, p-value=0.02622, beta=0.2064 in IVGTT-Based Insulin Secretion GWAS
Insulin sensitivity adj BMI, p-value=0.02235, beta=0.1142 in GENESIS GWAS
Matsuda insulin sensitivity index (ISI), p-value=0.03244, beta=0.13 in MAGIC insulin secretion during OGTT GW[illegible]
Insulin secretion rate, p-value=0.02634, beta=-0.1634 in IVGTT-Based Insulin Secretion GWAS
Type 2 diabetes adj BMI, p-value=0.098, OR=1.3231 in several datasets
Insulin sensitivity adj BMI, p-value=0.01076, beta=0.0938 in GENESIS GWAS

Table 2: Primary outcome and models used in replication analyses

**Cohort**
Metformin Genetics Consortium (MetGen)
Diabetes Prevention Program
The Study to Understand the Genetics of the Acute Response to Metformin and Glipizide in Humans

Reference

10
22

23

Participant characterestics

Type 2 diabetes
Prediabetes

Risk factors for type 2 diabetes; Medication naïve

Sample size

7,812
1,763

807

Primary outcome

Change in HbA1C
Diabetes incidence

Acute change in fasting glucose adjusting for baseline glucose

Covariates

baseline HbA1c, adherence, metformin dose, creatinine clearance and treatment scheme (whether on metformin monotherapy or dual therapy of metformin add-on to sulfonylureas) and the first 10 principal components (PCs)

age, sex, and the first 10 PCs

age, sex,body mass index and the first 10 PCs.

Table 3: Results of replication analyses

#CHROM	BEG	END	MARKER_ID	Effect_allele
5	149321776	149321776	chr5:149321776:C:T	T
4	129436566	129436566	chr4:129436566:C:T	T
6	24058036	24058036	chr6:24058036:C:G	G
10	115789989	115789989	chr10:115789989:A	G
7	53995198	53995198	chr7:53995198:C:G	G
2	179285708	179285708	chr2:179285708:C:T	T
6	104958449	104958449	chr6:104958449:C:T	T
8	43596695	43596695	chr8:43596695:C:T	T
16	12539602	12539602	chr16:12539602:C:T	T
11	37327400	37327400	chr11:37327400:T:C	C

P value	Hazard ratio (HR) for metformin failure	HR lower 95% CI
1.65E-07	0.42	0.3
1.83E-07	0.5	0.39
2.39E-07	0.4	0.29
5.79E-07	0.46	0.34
7.35E-07	0.5	0.39
8.32E-07	0.53	0.41
9.55E-07	0.49	0.36
1.17E-06	0.44	0.32
1.62E-06	0.52	0.4
1.87E-06	0.48	0.36

			TOD
**HR upper 95% CI**	**MAF**	**rs#**	
0.58	0.0625	rs10040292	
0.65	0.1062	rs9996721	
0.57	0.0546	rs9358746	
0.62	0.0685	rs76195229	
0.66	0.0982	rs4947856	
0.68	0.1687	rs1388182	
0.65	0.0734	rs76236717	
0.62	0.0635	rs115911409	
0.68	0.0942	rs11645654	
0.65	0.0883	rs4641440	

DAY STUDY

Nearest gene
AFAP1L1 intron
Close to C4orf33
Close to NRSN1
ATRNL1 intron (Interacts with MC4R)
Close to VSTM2A
Close to SESTD1
LIN28B intron
Close to POTEA
SNX29 intron
Close to IFTAP

Top associated trait on T2D knowledge portal

Waist-hip ratio adj BMI, p-value=8.67e-6, beta=-0.0414 in GIANT-UK Biobank GWAS Meta-analysis

Urinary sodium excretion, p-value=0.0003, beta=-0.0064 in UK Biobank urinary sodium and potassium excretion

Iron deficiency, p-value=0.001093, OR=1.1923 in GERA additive model age-related GWAS

Creatine kinase, p-value=0.02407, beta=-0.0467 in several datasets

eGFR-cys (serum cystatin C), p-value=0.000486, beta=-0.0134 in CKDGen 1000G GWAS-eGFRcys associations

Left ventricular ejection fraction, p-value=0.0046, beta=-0.0445 in UK Biobank Cardiac MRI LV GWAS

Heart failure, p-value=0.007144, OR=1.0364 in several datasets

Uric acid, p-value=0.0004135, beta=-0.1416 in Middle Eastern Qatari population WGS

Coronary artery disease, p-value=0.0008661, OR=0.9774 in several datasets

Serum albumin, p-value=0.03318, beta=0.0347 in several datasets

Related trait on portal

Insulin sensitivity, p-value=0.005159, beta=0.4433 in GENESIS GWAS

Fasting insulin adj BMI, p-value=0.05971, beta=0.0242 in several datasets

Disposition index adj BMI, p-value=0.05173, beta=-0.074 in IVGTT-Based Insulin Secretion GWAS

Disposition index adj BMI, p-value=0.02622, beta=0.2064 in IVGTT-Based Insulin Secretion GWAS

Insulin sensitivity adj BMI, p-value=0.02235, beta=0.1142 in GENESIS GWAS

Matsuda insulin sensitivity index (ISI), p-value=0.03244, beta=0.13 in MAGIC insulin secretion during OGTT GV

Insulin secretion rate, p-value=0.02634, beta=-0.1634 in IVGTT-Based Insulin Secretion GWAS

Type 2 diabetes adj BMI, p-value=0.098, OR=1.3231 in several datasets

Insulin sensitivity adj BMI, p-value=0.01076, beta=0.0938 in GENESIS GWAS

Adiponectin, p-value=0.064, beta=1.8545 in several datasets

			SUGA
**allele A**	**allele B (effect allele)**	**MAF**	***P* value**
C	T	0.049997	0.98261
C	T	0.131031	0.888713
C	G	0.0705824	0.818468
A	G	0.0404802	0.075652
C	G	0.0875415	0.491574
C	T	0.0926623	0.969815
C	T	0.0971908	0.199871
C	T	0.0343333	0.125714
C	T	0.132012	0.544732
T	C	0.0366629	0.427043

AR-MGH

Beta (effect size)	Direction of effect in relation to TODAY
-0.00136047	Same
-0.00651633	Same
-0.014083	Same
0.137797	Opposite
-0.0385151	Same
-0.00195398	Same
-0.0679398	Same
-0.129499	Same
-0.0280938	Same
-0.0596888	Same

A2	A1 (effect allele)	MAF	Beta (Effect size)	Standard error	*P* value
C	T	0.037424	-0.03828	0	9.52E-01
C	T	0.126491	0.03971	0	9.12E-01
C	G	0.07539	-0.1397	0	7.43E-01
A	G	0.032838	0.4519	0	4.83E-01
C	G	0.08149	0.1491	0	7.34E-01
C	T	0.103482	0.4007	0	2.45E-01
C	T	0.084484	-0.7016	0	1.34E-01
C	T	0.030653	-0.7751	0	2.60E-01
C	T	0.122047	-0.2651	0	4.68E-01
T	C	0.043592	-0.08805	0	8.66E-01

DPP		
**HR (95% CI)**	***P* value for GxE**	**Imputation info score**
0.9624 (0.274-3.381)	9.45E-01	0.939
1.041 (0.5147-2.103)	8.38E-01	0.909
0.8696 (0.3779-2.001)	7.01E-01	0.992
1.571 (0.4447-5.552)	3.90E-01	0.95
1.161 (0.4914-2.742)	8.81E-01	0.925
1.493 (0.7599-2.933)	4.16E-01	0.838
0.4958 (0.198-1.241)	8.82E-02	0.954
0.4607 (0.1195-1.776)	3.77E-01	0.993
0.7671 (0.3752-1.568)	4.27E-01	0.994
0.9157 (0.3289-2.55)	6.90E-01	0.956

Direction of effect in relation to TODA	RSID	Chr:position
Same	rs10040292	5: 148701339
Opposite	rs9996721	4: 130357721
Same	rs9358746	6 24058264
Opposite	**rs76195229**	**10 117549500**
Opposite	rs4947856	7 54062891
Opposite	rs1388182	2 180150435
Same	rs76236717	6 105406324
Same	rs115911409	8 43451838
Same	rs11645654	16 12633459
Same	rs4641440	11 37348950

	METGEN		
**Effect allele**	**Non-effect allele**	**EAF**	**Samples available**
T	C	0.009	8316
T	C	0.144	10519
G	C	0.071	12576
**G**	**A**	**0.004**	**7048**
G	C	0.085	10519
T	C	0.055	12582
T	C	0.106	12581
T	C	0.005	7048
T	C	0.162	12581
C	T	0.002	7048

Beta ± SE	*P* value
-0.078 ± 0.082	0.34
0.01 ± 0.018	0.58
0.008 ± 0.022	0.71
**0.336 ± 0.125**	**0.007**
0.0112 ± 0.02	0.56
0.01 ± 0.025	0.67
-0.02 ± 0.018	0.27
0.099 ± 0.142	0.49
-0.001 ± 0.015	0.94
0.235 ± 0.223	0.29

Table 4: Lookup of variants relevant to metformin response in adults and metformin transport

SNP	Position	Nearest gene	Effect_allele
rs8192675	3:171007094	*SLC2A2*	C
rs3889348	7:5298844	*SLC29A4*	G
rs2162145	8:67747912	*CPA6*	T
rs11212617	11:108412434	*ATM*	C
rs254271	19:54127382	*PRPF31*	C
rs202220802	6:160139848	*SLC22A1*	
rs12208357	6:160122116	*SLC22A1*	
rs12943590	17:19716685	*SLC47A2*	
rs2076828	6:160451754	*SLC22A3*	
rs2289669	17:19560030	*SLC47A1*	
rs272893	5:132327369	*SLC22A4*	
rs316019	6:160249250	*SLC22A2*	
rs34130495	6:160139792	*SLC22A1*	
rs622342	6:160151834	*SLC22A1*	
rs8065082	17:19561878	*SLC47A1*	

Non_effect_allele	MAF	Direction of metformin response
T	0.4821	better
A	0.2798	worse
C	0.4514	better
A	0.4821	better
G	0.2966	worse
	0.1508	
	0.0268	
	0.2748	
	0.3819	
	0.3155	
	0.3244	
	0.0913	
	0.0109	
	0.2887	
	0.4355	

Hazard ratio	*P* value
1.03	0.75
1.05	0.63
0.9	0.29
1.04	0.7
0.898	0.28
0.97	0.81
0.83	0.49
0.999	0.99
0.98	0.84
1.12	0.28
0.99	0.88
0.85	0.28
1.23	0.69
1.12	0.29
1.03	0.71

Direction of effect in relation to TODAY
opposite
opposite
same
same
opposite

Table 5: Quality control tests using partitioned polygenic scores (pPS) on metabolic traits

	Trait~Cluster risk score+age+sex+PC1-10			
Cluster	*Beta cell*	*Proinsulin*	*Obesity*	*Lipodystrophy*
Baseline log10 triglyceride				
β	-0.00075	-0.0064	0.0019	0.0013
P value	0.68	0.16	0.44	0.62
Baseline log10 fasting insulin				
β	-0.0043	-0.0029	0.0026	0.0034
P value	0.067	0.61	0.42	0.29
Baseline HDL				
β	0.021	-0.057	0.098	-0.091
P value	0.77	0.75	0.31	0.35
Baseline BMIZ				
β	0.00082	0.015	0.0087	0.0052
p-value	0.83	0.11	0.089	0.31

Liver/lipid

-0.0027
0.35

0.0044
0.25

0.032
0.78

0.011
0.07

Table 6- Results of pPS analysis

**Association of baseline traits with cluster genetic scores**		
Baseline traits (sometimes natual-log-transformed)~Cluster risk score (CRS)+a		
**Cluster**	*Beta cell*	*Proinsulin*
ln(Fasting glucose baseline)		
β	0.0018	0.00021
P value	0.27	0.96
ln(Fasting insulin baseline)		
β	-0.0097	-0.0066
P value	0.066	0.62
ln(insulin sensiitivity index), 1/Fasting insulin		
β	0.009	0.0066
P value	0.1	0.62
ln(Insulinogenic index),△I30/△G30		
β	-0.02	0.0071
P value	**0.02**	0.74
ln(C peptide index) △C30/△G30		
β	-0.013	0.0023
P value	0.075	0.9
ln(oral disposition index), 1/IF × △I30/△G30 and 1/IF × △C30/△G30		
β	-0.0054	0.011
P value	0.49	0.56
C-peptide baseline		
β	-0.026	-0.02
P value	**0.047**	0.54

ge+sex+PC1-3+rosig+lifestyle

Obesity	*Lipodystrophy*	*Liver/lipid*
-0.0027	0.0025	0.001
0.23	0.27	0.7
0.0064	0.0081	0.013
0.38	0.26	0.13
-0.0071	-0.0088	-0.015
0.34	0.24	0.092
0.021	-0.0036	0.0095
0.066	0.76	0.5
0.011	0.0063	0.014
0.24	0.51	0.24
0.014	-0.00073	-0.0064
0.18	0.94	0.6
0.0066	0.037	0.05
0.71	**0.039**	**0.019**

Association of 6 months change in traits with cluster genetic scores

delta traits~CRS+baseline traits(sometimes natura

**Cluster**	*Beta cell*
ΔFasting glucose	
β	-0.41
P value	0.11
ΔFasting insulin	
β	-0.086
P value	0.65
Δinsulin sensitivity index, 1/Fasting insulin	
β	0.00025
P value	0.52
ΔInsulinogenic index, △I30/△G30	
β	0.0092
P value	0.71
ΔC peptide index, △C30/△G30	
β	-0.0011
P value	0.42
Δoral disposition index, 1/IF × △I30/△G30 and 1/IF × △C30/△G30	
β	5.48E-05
P value	0.96
ΔC-peptide	
β	-0.022
P value	0.11

al-log-transformed)+age+sex+PC1-3+rosig+lifestyle

Proinsulin	*Obesity*	*Lipodystrophy*	*Liver/lipid*
-0.093	-0.26	-0.18	-0.11
0.88	0.45	0.59	0.8
-0.28	-0.35	-0.21	-0.075
0.54	0.17	0.41	0.81
0.00052	-0.00045	-0.00048	-0.0006
0.57	0.38	0.35	0.34
0.0048	-0.04	0.0017	0.054
0.94	0.22	0.96	0.17
0.00075	0.00037	0.0022	0.0033
0.82	0.84	0.21	0.13
0.00051	-0.0019	0.00048	0.0034
0.86	0.22	0.76	0.067
-0.039	-0.016	-0.016	0.015
0.26	0.41	0.39	0.51

Metformin survival test on cluster genetic scores

h(t)~h(t0)*exp(beta*CRS+beta*AGEBASE+beta*sex+beta*PC1-3+b[illegible]

**Cluster**	*Beta cell*	*Proinsulin*	*Obesity*
HR	0.995	0.99	1.02
P value	0.7	0.76	0.33

eta*Rosig+beta*Lifestyle)

Lipodystrophy	*Liver/lipid*
1.02	0.99
0.22	0.68
